# Using cultural historical activity theory to understand how post-graduate residents perform discharge planning at a medical center in Taiwan

**DOI:** 10.1186/s12909-023-05003-8

**Published:** 2024-01-26

**Authors:** Fang- Yih Liaw, Yaw-Wen Chang, Po-Fang Tsai

**Affiliations:** 1 Department of Family and Community Medicine, Tri-Service General Hospital, National Defense Medical Center, Taipei, Taiwan; 2 Graduate Institute of Humanities in Medicine, College of Humanities and Social Sciences, Taipei Medical University, Taipei, Taiwan; 3 School of Medicine, College of Life Sciences and Medicine, National Tsing Hua University, No. 101, Section 2, Kuang-Fu Road, Hsinchu City, 300044 Taiwan

**Keywords:** Cultural-historical activity theory, Post-graduate year, Discharge planning education

## Abstract

**Background:**

Despite the importance of discharge planning in physicians’ education, currently in most countries, no identical training is provided. Difficulties in promoting physician discharge planning education in Taiwan are still noted. This study aims to find the physicians’ role of discharge planning training in educating post graduate year residents (PGY) in Taiwan.

**Materials and methods:**

We took advantage of government and hospital policies that promote the discharge planning program to teach and implement it, beginning with PGY residents by incorporating it into their training program. We recruited 30 PGY residents who were attending their three-month general internal medicine training from 2018 to 2019. They were interviewed at the end of the program using cultural-historical activity theory (CHAT). Qualitative research methods were used to further understand how discharge planning and care was implemented.

**Results:**

Trainees initially believed that they did not have any role in discharge planning. Using the cycle of expansive learning, we found that the role of physicians in discharge planning was unclear. There were still some inconsistencies in the teaching and implementation of the discharge planning program for PGY residents that needed to be resolved, but this study also let participants learn through practice to improve their identification of discharge planning.

**Conclusions:**

This study analyzed the impact of a discharge planning program for PGY physicians in Taiwan. It showed that the program affected physicians’ practice and medical education, although some contradictions remain.

## Practice points

1. For healthcare institutions, we suggest review both the inconsistences and advantages of discharge planning (DP) in practice, so that either decreasing the former or enhancing the latter would benefit the institutional reform of DP. Du to the population density in Taiwan, our case study may be applicable only to international metropolises with similar population densities, rather than other regions within the same country.
2. For policymakers, we suggest pay more attention and invest more resources to DP in order to keep the continuity of care between inpatients and patients in communities, care centers or at home at an aged society. We believe that this recommendation will be more beneficial for societies with population densities lower than that of Taiwan.
3. For medical educators, we suggest the essence of DP in PGY training through which they understand the importance of continuity of care and teamwork, increase their self-identification as physicians, and are educated as the next generation of physicians on the concept and actual practice of discharge planning and care. We also suggest the usefulness of Cultural-historical activity theory as a methodological framework for practice-based learning in intricate learning environments, for medical educators in Taiwan and in global.

## Background

Discharge planning is crucial for the continuity of care as it ensures a smooth transition for patients from the hospital to their next level of care, which could be home, a day care center, or a nursing home. It takes a holistic view of the patient's health and individual needs, and often involves setting up referrals with supportive organizations in the community, such as occupational therapists, primary care providers, and home health care. An effective discharge planning can also rise the quality of care through reducing patient’s readmissions. Although discharge planning is essential in the continuity of care, there is no consistent standard of practice or training worldwide [1]. Discharge planning is a service provided by a team that includes physicians. Physicians regard discharge planning as important, but some barriers exist [2], causing discontinuity in the patient’s care after discharge.

Although a formal curriculum in discharge planning is needed [3], formal instruction in discharge planning is limited in most residency programs [1, 4]. Residents in the U.S.A. had reported a lack of understanding of who is responsible for discharge education [5].

In Taiwan, we faced similar difficulties. Although the Taiwanese government began a subsidy program for long-term care from 2005, hospitals may implement varying discharge planning standards, and it is not possible to always have a dedicated discharge planner. Taking the Doctor’s opinion paper (DOP), a key document which might issue after the comprehensive assessment of discharge planning, as an example, Taiwan government amended the Long-Term Care Services Act to confine the irreplaceability of DOP with discharge summary or certificate of diagnosis in 2019.

Besides, the complicated discharge planning education for residents can vary depending on their coworkers, patient comorbidities, patient census, and established local practices regarding hospital discharge [4]. For example, the unclear division of responsibilities between discharge planners and primary care physicians may cause difficulties in team communication and lead to the discontinuity of patient care after discharge. As a result, in the discharge planning process, physicians passively wait for discharge planners’ instructions and are unclear about the subsequent phases of care [2, 5].

The discharge process can be taught during residency training [6]. In this study, we emphasized that we should make efforts to teach such skills and focus on improving education on hospital discharges during the post-graduate year (PGY).

Before SARS occurred in 2003, premature specialization and inadequate basic training of physicians resulted in overwork and accidents in Taiwan. Then PGY general medical training was implemented, which required graduates to receive general medical residency training at a government-accredited hospital before assuming residency in a specialty [7]. Since 2003, the Taiwan Medical Accreditation Council instituted a comprehensive Postgraduate Year (PGY) training system, requiring a three-month duration after completion of undergraduate medical training. Emphasizing continuity and holistic care, PGY residents were expected to adopt a holistic care mindset prior to embarking on specialist physician training. Subsequently, as of 2013, the medical education program in Taiwan has evolved to include a full 2-year PGY residency program immediately following undergraduate medical training. While the core concept of holistic care must be learned during PGY training, discharge planning was incorporated into PGY education in the recent years.

Discharge planning training is most appropriate for PGY training and can change the actions of future physicians. However, barriers, such as physicians’ passive attitude, are constantly being identified. We should correct such barriers in teaching and practice and activity theory was used to approach.

### Cultural-historical activity theory

Cultural-historical activity theory (CHAT), also known as Activity Theory (AT), holds that human activity can be described and analyzed, and that all activities have a structure, happen under certain conditions, and can be assisted by particular tools. In its original iteration, Activity Theory comprises three fundamental principles: the subject, representing the individual performing the activity; the object, denoting the objective of the activity; the artifacts, signifying the tools utilized to accomplish the objective. Subsequently, scholars have introduced numerous additional factors to enhance and expand upon this theoretical model, such as rules, communities, and division of labor [8, 9]. In addition to analyzing Technology-Enhanced Learning and examining cultural activities at work, investigating professional work practices is also one of CHAT or AT’s practical applications.

In the activity theory, transformations are not viewed as linear movement toward pre-fixed end points. They are understood as zones of proximal development (ZPD). ZPD is a variable space between what the learner has mastered and what they cannot yet do. It is a well-known concept in education originally proposed by Vygotsky [8]. It depends on the individual being able to adequately perceive what is actionable when getting help.

Engeström refined the zones of proximal development (ZPD) in 2015 based on collective learning and development. ZPD is in the middle of the present and the future. Teach learners in the ZPD and help them to reach independence can be a solution to the contradictions [9]. Engestrom mentioned that nowadays fragmentation of medical education and patient care has been noted, but more within patients with long term conditions. Therefore, we need to expand the object of medicine and broader collaborative and interdisciplinary approaches from medical practitioners and organizations [9].

Physicians take a passive role in learning discharge planning and are unaware of their importance to the discharge planning team in Taiwan. Therefore, we are currently in a ZPD concerning this issue; moreover, we might be in such a condition until we solve existing contradictions through the PGY curriculum and hands-on work.

**Fig. 1:**
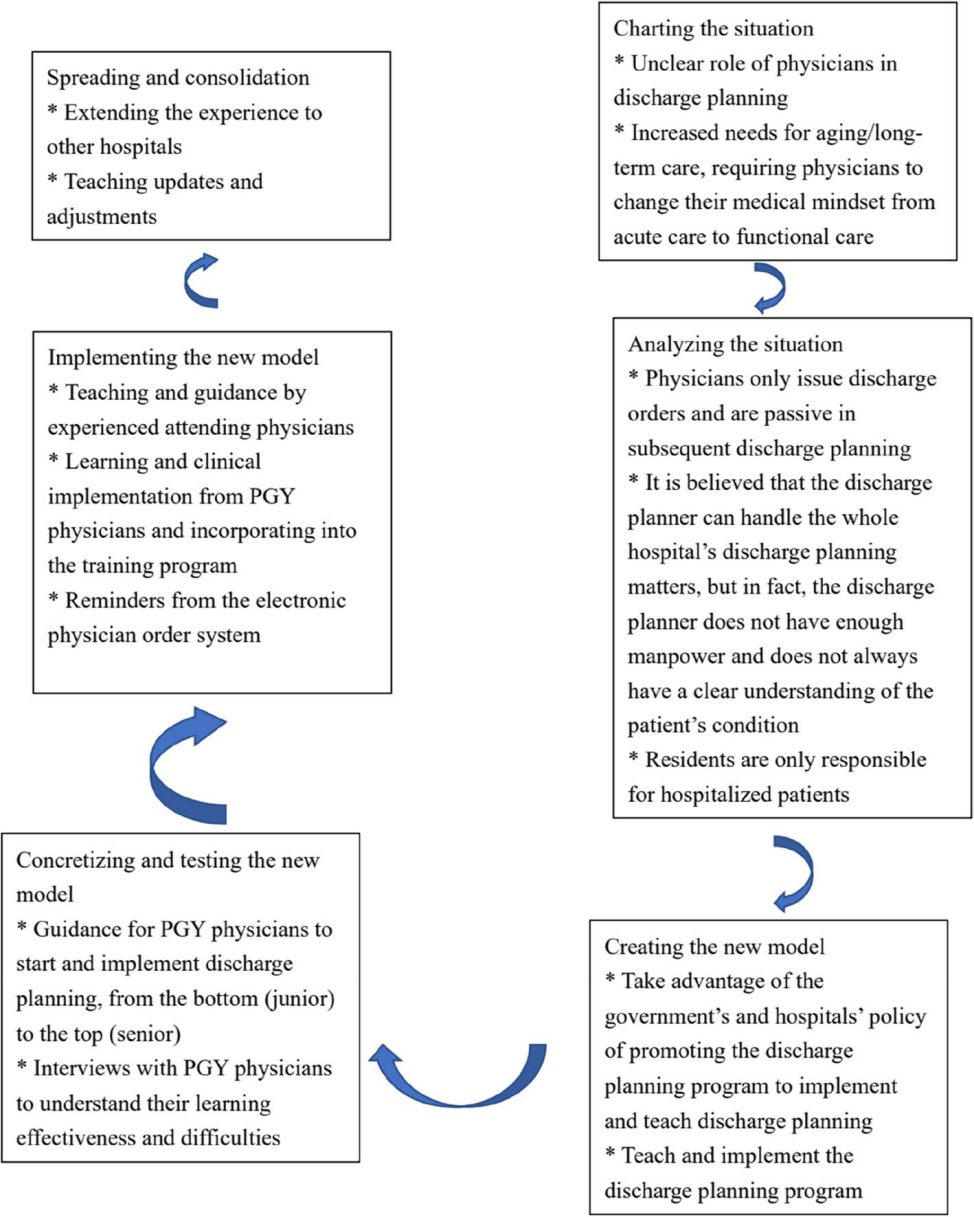
Steps of expansive learning of discharge planning curriculum

Expansive learning provides a way of analyzing and promoting learning for change. The theory of expansive learning focuses on the learning of new patterns of activity that are not yet there; they learn by design. The subjects of expansive learning are involved in multilevel learning process [10]. Expansive learning proceeds by learning actions that form cycles of expansive learning (Fig. 1).

In short, CHAT offers historically formed systemic contradictions as a starting point for analysis and can be used to form a working hypothesis about the pervasive contradictions of medical work [11]. It assumes that learning occurs through practice and creates new forms of knowledge and activity. It can extend learning and is being increasingly used in medical education scenarios.

This study will examine the alignment of physician education and implementation of patient discharge planning. We also discuss the training and impact of current discharge planning programs on PGY physicians in Taiwan using CHAT.

## Methods

### Study context

The discharge planning program incorporates principles of discharge planning into the clinical curriculum. It was developed and implemented in the medical center of PGY clinical curriculum of internal medicine in 2018–2019.

### Participants

During the 2018–2019 academic year, 30 PGY residents participated in the discharge planning program: 23 men and 7 women, aged 27 to 30, from North Taiwan, were rotated on the service. To study the program, after completing three-month general internal medicine training, they were invited to participate in interviews for an average of 50 min, and were informed of their options to accept or refuse.

### Curriculum

During the three months of general internal medicine training, PGY residents are required to attend courses that emphasize the importance of discharge planning by a family medical physician. The lecture was for 2 h, including introduction to the rest of the team, why the role of the physician is important, and the responsibilities of the physician.

The curriculum also introduces the implementation of state-mandated discharge planning, which includes (1) identifying patients with discharge planning needs, (2) initiating team-wide meetings and documenting discharge planning, and (3) post-discharge telephone follow-up and documentation.

### Clinical implementation

PGY residents are required to perform discharge planning when they learn of the needs of the patients they care for during their training in general internal medicine. The electronic physician order system helped and directed physicians to initiate team-wide discharge meetings to support patient follow-up when a patient is admitted to the hospital and may need discharge planning.

By the end of their training in General Internal Medicine, PGY residents must complete at least three discharge planning cases, including initiation and documentation of team-wide discharge planning meetings and post-discharge follow-up telephone visits as their completed assignment for PGY training in Internal Medicine.

The implementation of the program across the PGY offered the opportunity to investigate how discharge planning is incorporated into clinical practice.

### The cycle of expansive learning

In the cycle of expansive learning (Fig. 1), we found that the role of physicians in discharge planning is unclear, and the need for aging care and long-term care is increasing, requiring physicians to change their medical mindset from acute to functional care. By using government and hospital policies to promote the discharge planning program, we began to teach and implement the discharge planning program. Learning and clinical implementation began with PGY physicians and were incorporated into the training program. We also engaged experienced senior physicians to teach and guide this program, and we enhanced the electronic physician instruction system to provide reminders.

### Coding and analysis

Two investigators (FYL and PFT) coded the PGY interviews and monitored the coding process. We borrowed from the constructivist approach of grounded theory and used open coding to analyze and explore initial ideas. These themes were examined at a conceptual level to explore interrelationships.

We collated and reconciled themes through group discussion to form a coding structure. Two authors with humanities and social sciences background designed the study. Another two authors with MD background collected the data in clinical setting. One faculty FYL, as both researcher and facilitator, led the lectures.

As far as the aim of the study, the sample specificity, and quality of dialogue are concerned, the concept of “information power” would be a greater guide which satisfied with our simple size (*n* = 30) than the conventional concept of “data saturation” from the grounded theory approach [12].

MAXQDA, a cloud-based qualitative data management program, was used to support data analysis. As we analyzed our data, we use CHAT as notable explanatory power for the patterns we identified.

## Results

Based on interviews with PGY participants about learning discharge planning after completing the curriculum and performing actual cases, the following findings emerged from this study. We first discussed the elements of activity theories, then indicated the four contradictions, and found four PGY’s improvements in the end. We describe each theme below along with sample supporting quotes in Tables 1 and 2.

**Table 1:** Themes and illustrative quotes from contradictions

(1) Disagreements across communities	Disagreements across communities
	***During class, we inform residents that they can ask the attending physician in the discharge planning program for help if they face difficulties with discharge planning for patients. Sometimes, when a resident feels that a patient needs additional help, but the attending physician does not think the same, the resident wonders if they are overthinking and overextending themselves to help their patients. (PGY20)***
	Disagreements in communication with caregivers
	***Another challenge in implementation is that sometimes the caregiver, who may not be a family member, may be difficult to reach. The person who should be taught is often absent, which is sometimes rather discouraging. (PGY13)***
(2) Subspecialty training	Not providing comprehensive patient care due to subspecialty training
	***You may want to know the status of a patient after discharge, but there are many others. Sometimes, you move to another department, but the patients in the previous department are still waiting for your follow-up. If you were called two months later, you would not remember how the patient was doing*** ***You won’t necessarily know if a patient is readmitted. Even if you know that they are admitted, they are not under your care, so I think there is a difference. (PGY23)***
	Residents are only responsible for hospitalization
	***Residents are only responsible for hospitalization and should focus on medical care. (PGY22)***
3) Daily work overload	***The patient can be discharged the same day but because you unconsciously postpone this, the patient may already be discharged when you finish priority tasks, or it may be too late in the day. (PGY5)*** ***Residents currently have to take care of these matters, which is too exhausting. The doctor is overwhelmed with the care of the patients. (PGY8)*** ***The implementation of discharge planning by our residents is indeed helpful to the patients, but we still experience it as an overload. (PGY16)***

**Table 2:** Themes and illustrative quotes from participants improvement

1. Identification of discharge planning needs	***First, we need to determine whether patients are able to perform our nursing techniques on their own after discharge. Second, if patients have problems after getting discharged because the doctor’s orders were not followed or the issue was not solved, they can ask for help. (PGY20)***
2. Self-identification	***I think discharge planning is a good idea because patients really need this service. It involves many tasks. Following up with patients after discharge is good for the patients, but it is also an opportunity for me to learn and grow. (PGY27)*** ***Helping doctors depends, in part, on the situation. I am a relatively inexperienced physician, and it enhances our learning to understand a patient's discharge process, that is, the difficulties encountered during discharge and resources available. With the help of the discharge planning team, I learn more details. (PGY25)***
3. Improving division of labor and the relationship between team members	***The division of labor across different roles is quite helpful to our medical care team, and their advice tends to be more comprehensive. (PGY18)***
4. Improving the physician–patient relationship (discharge follow-up)	***We need to understand the changes in the patient’s condition and listen to what is bothering them. If we spend more time communicating with them, patient satisfaction and comfort will improve. The more the patients know, the safer they will feel. (PGY18)***

### The elements of CHAT

**Fig. 2:**
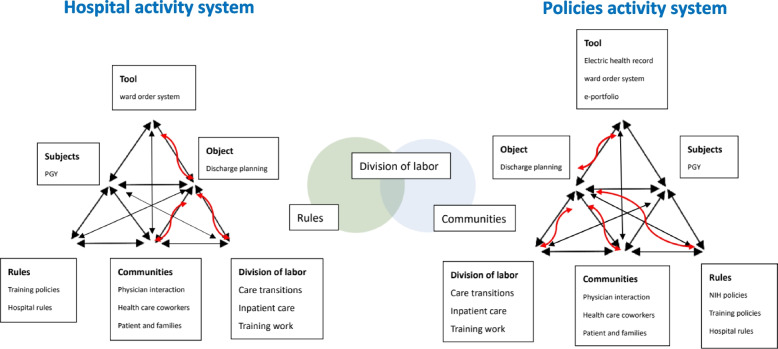
Systemic contradictions as sources of discharge planning training for PGY

In Fig. 2, this study discusses the elements of CHAT, including communities: disagreements between communities or communication with caregivers; division of labor: daily distribution of work, occasional inability to focus on stable patients being discharged; rules: unable to provide comprehensive patient care due to subspecialty training; and tools: additional time spent following up with patients by phone and completing electronic team medical records.

Furthermore, we can find that division of daily work can be positively integrated by using the rules of the national policy and communities of team members (in the middle of Fig. 2).

#### Communities: differences of opinion across communities

The primary supervisor is the attending physician, who is also the primary decision maker for any disposition or treatment for a patient. Thus, the attitude of the attending physician has a major impact on PGY participants. Conflicts may also arise, but PGY participants respect their teachers and keep these doubts and conflicts to themselves. These problems gradually emerged during the implementation of discharge planning. Since it is a national and hospital policy, physicians tend to implement it and communicate with each other.

S1 Communities: communication with caregivers.

In medical care, “total care” unconsciously deprives patients and their families of the opportunity to learn how to care, causing patients to depend on hospitals. In addition to the guidance by medical staff, the patient’s self-care and the level of commitment of the family are also important in the follow-up care, which requires two-way communication. This explains why the attitude of the patient and the family are also important.

#### Division of labor: daily distribution of work, sometimes being unable to focus on stable patients to be discharged

The main reason discharge planning is difficult to implement in clinical practice is the field of physician training. Although discharge planning is important, the residents only care about hospitalized patients as the patients to be discharged are typically in stable condition. As a result, it is only natural that the priorities of daily work shift, as in other countries and professions. If the curriculum teaches PGY physicians why discharge planning is necessary and their role in it, it strengthens their self-identification. It takes less time, but improves the quality of care at discharge.

#### Rules: being unable to provide comprehensive patient care because of subspecialty training

Discontinuity of patient care caused by subspecialty training is a difficulty encountered in today’s medical education. Greysen and colleagues also found PGYs particularly focused on events occurring in the hospital and described follow-up care as beyond their routine care [4]. As learning and care takes place in the hospital, communication with subsequent caregivers is also a major challenge when the patient is discharged.

#### Tools: additional time required to follow up with patients by phone and complete electronic team medical records

While discharge planning improves the quality of patient care and increases the resident's ability to discharge plan, it also increases physician workload and causes discomfort for some physicians. While telephone follow-up allows residents to keep track of their patients’ status after discharge, it also requires residents to spend additional time calling patients at specific locations. Sometimes, because of their busy schedules, they forget to keep track of their patients and feel stressed. Therefore, the curriculum can be further adjusted in the future. This is the same situation in all other studies.

### Contradiction

There are still some inconsistencies in the teaching and implementation of the discharge planning program for PGY participants that need to be resolved: (1) disagreements across communities or in communication with caregivers; (2) feeling overburdened due to the day-to-day work distribution; sometimes not being able to focus on stable patients being discharged (3) worrying about lack of experience and not providing comprehensive patient care due to subspecialty training; and (4) residents only feeling responsible for hospitalization; they did not like to spend additional time following up with patients by phone and completing electronic team medical records.

#### Differences of opinion across communities

The program initially targeted PGY participants, but their attending physicians in clinical training have not received the appropriate courses and training. Some still believe that discharge planning should be the sole responsibility of discharge planners. Some PGY participants felt that it was necessary to initiate a discharge planning team for a patient. However, the attending physician disagreed, leading to inconsistencies in the PGY participant’s implementation. If a PGY participant had problems with implementation and asked the attending physician, but did not receive an appropriate response, this could lead to problems and confusion in implementation.

This problem was partially solved by improving electronic physician orders. In the early stages of discharge planning implementation, some physicians are not quite sure whether a patient needs discharge planning or there is no consensus among physicians. If the patient is eligible for follow-up after hospitalization, the physician is advised that discharge planning for that patient should be initiated through the electronic physician order system. Motamedi and colleagues had reviewed that computer-enabled discharge communications appear beneficial and improve timeliness and physicians’ satisfaction [13].

When the physician initiates the team’s plan of care, team members can provide ratings and recommendations through the electronic medical record system. The physician can then discuss and develop the patient’s discharge plan based on the team’s recommendations. Using system reminders and team recommendations, PGY participants could more confidently discuss their patients’ discharge plans with their teachers.

##### Communication with caregivers

Although the physician–patient relationship has been enhanced, the patient’s primary caregiver may change after discharge, and there are concerns about the subsequent care. A patient’s caregiver while in the hospital could be replaced by someone else after discharge. This can lead to discontinuity in patient follow-up and make PGY participants feel powerless. They believe that even if they give good discharge care instructions, these efforts will be for naught if subsequent implementation fails.

#### Feeling overburdened

In the PGY training phase, residents have many policy-mandated assignments in addition to their clinical work. At this point, discharge planning seems to be an additional burden on the residents.

Although the physician should make discharge-related medical orders, it is necessary for discharge planning to retain the discussions and records of team meetings. PGY residents must record the minutes of the meetings and keep them in the nursing system. After the patient is discharged from the hospital, the resident must conduct follow-up meetings and record them. Residents in PGY have to make phone calls during office hours to inquire about patients' discharge status, which puts a lot of stress on them. Inpatient medical issues are complicated, and physicians must prioritize them. Patients who are ready to be discharged or who are discharged in a stable condition are given lower priority.

#### Worried about the lack of experience

As a direct transition from medical school to residency, PGY participants are the youngest residents in the hospital, and some may still need to adjust. During the training on discharge planning, some participants felt that implementation was somewhat difficult because they lacked experience, for example, insufficient familiarity with other team members’ work. However, some participants felt that learning these things in the PGY phase was consistent with the concept of holistic care. In particular, when the workload of attendings or residents is much heavier and they are not available to do discharge planning, PGY participants need to step in to do it under the guidance of other attendings.

##### Unable to provide comprehensive patient care because of subspecialty training

Residents are still in the training phase and have to change internal medicine departments every month and see patients at other attending physicians, so they cannot follow the continuing care of their patients. They cannot provide continuous and comprehensive care. However, this problem is present in every residency training system.

#### Residents only responsible for hospitalization

Some residents feel overworked when caring for their patients and are frustrated with the extra work they have to do during training, as they are caring for patients and also attending classes. They believe that residents only need to take care of the patients in the hospital. Patients will return to the attending physician's clinic for follow-up after discharge, and residents do not need to know the discharge status of patients. If a patient has a problem after discharge, medical care in Taiwan is convenient, accessible and inexpensive. The patient can return to the hospital and be readmitted for treatment at any time if necessary.

##### Additional time required to follow up with patients by phone and complete electronic team medical records

Discharge planning requires a team meeting to be documented by the PGY physician and a telephone call to follow up and document the patient’s status at discharge. Initially, telephone follow-ups were documented on paper, but later they were added to the electronic medical record system. However, physicians feel that these records take additional time on top of the daily records.

### Participants’ improvement

Participants learn through practice to improve (1) their identification of discharge planning needs, (2) their self-identification within the team, (3) the relationship between team members, and (4) the physician–patient relationship.

#### Improving identification of discharge planning needs

Interviews revealed that residents understood, based on their training, why discharge planning is necessary, how important it is to patients, and the physician’s role in it. The structure and process developed to remind PGY participants also helped them identify their patients’ needs, improve communication and rapport with team members, contribute ideas, plan post-discharge care during hospitalization, and improve the quality of patient care. Participants felt that their ability to identify patients’ needs at discharge had improved significantly, which was not the case during their internship. They used to do what the physician told them to do without applying their ideas, but after the classes and practical work, they gradually became more aware of the patient’s discharge planning needs.

#### Improving self-identification

The discharge planning program is sponsored and implemented by Taiwan’s Ministry of Health and Welfare to improve the quality of long-term care. Because it is a government-sponsored program and is included in hospital accreditation, hospitals must implement the program and review and improve the number of patients requiring discharge planning each month. Through the curriculum and hands-on implementation of discharge planning, PGY participants became better integrated into the team, and their self-identification with the physicians’ role in discharge planning was strengthened. This program can create an encouraging atmosphere in hospitals. As team leaders, physicians are more aware of and dedicated to implementing discharge planning.

Self-reporting by physicians also increased significantly. Discharge planners were assumed to be responsible for discharge planning. PGY’s daily care of patients is comprehensive and continuous. Physicians know more about patients’ conditions, functional status, and family and financial situations than discharge planners and nurses working three shifts. Within the hospital, residents are in the best position to understand their patients’ needs, more so than attending physicians. Residents in PGY recognize this and are more self-aware. They no longer rely on discharge planners or attending physicians and can initiate and plan care through the discharge planning team as soon as a patient needs discharge planning.

#### Improving division of labor and the relationship between team members (know more about other roles although passively)

Okoniewska et al. found that poor communication between care team members can delay patient readiness for discharge and subsequent discharge completion [2].

During the curriculum orientation, other members of the discharge planning team were introduced so that PGY participants knew the job duties of other team members.

PGY participants were required to initiate a discharge planning team meeting with at least two other members, including a pharmacist, dietitian, rehabilitation specialist, psychologist, and discharge planner, to implement the discharge planning process. During the implementation process, participants were able to better understand other roles and responsibilities. When discussing patient discharge needs, participants noted that all roles work together to help patients. Although they were forced to convene a discharge planning team meeting, participants were also more aware of the hospital's processes and discussed the best options for patient. In the past, physicians often believed that they only needed to contact a discharge planner to discharge their patients. This led to an unclear division of labor in discharge planning and inadequate quality of discharge planning for patients. After the three-month training, the participants became familiar with the team. They were able to actively discuss the needs of hospitalized patients with team members to improve the efficiency of the division of labor.

#### Improving the physician–patient relationship (discharge follow-up)

Miscommunication about the patient instructions and follow-up appointments can lead to patient dissatisfaction and delays in discharge [14]. Because of the discharge planning program, physicians must instruct patients on post-discharge care precautions, medication, etc. They must also conduct telephone follow-up visits after patients have been discharged to understand patients’ actual status after discharge. Telephone calls to patients after discharge from the hospital are confirmed as an important part of care transitions [15].

In the past, patients were returned to the attending physician’s office for follow-up, and residents often ended their care of patients after discharge and knew nothing about the patients’ condition after discharge. Phone calls allow residents to know how patients are doing after discharge and to really understand the importance of discharge planning for patients. They become more aware of the importance of discharge planning, and the residents’ connection to patients is more comprehensive and uninterrupted. PGY residents also care more about quality of patient care.

## Discussion

In this study, we evaluated discharge planning implementation of PGY in Taiwan. We also investigated the curriculum integrating government policy developed in clinical workplaces. Applying activity theory, two identified activity systems were involved in the process: the original system and the policy integrated teaching system. In the policy integrated teaching system, division of labor of daily work can be positively integrated by the rules of the national policy and communities of team members.

The findings further illustrate how the systems came together in interaction, including disagreements between communities; imbalance of division of labor; unable to provide comprehensive patient care due to rules; and unfriendly medical tools.

Our findings are on the important issue of methodological framework for finding contradictions of the physicians’ role in discharge planning, practice-based learning in intricate discharge planning learning environments, and taking advantage of national policies on how discharge planning with change in practice.

The inconsistencies in implementing the discharge planning program for the PGY include disagreements across communities, subspecialty training system, and daily work overload.

### Disagreements across communities

In Taiwan, despite the positive attitudes toward discharge planning, many physicians still demonstrate an unsatisfactory level of knowledge and behavior with regard to the same. To enhance the implementation of discharge planning, a standard evaluation procedure for interdisciplinary discharge planning and improved physician awareness concerning the importance of discharge planning are needed.

The author suggested that to enhance the awareness of the implementation of discharge planning, physicians should be made aware of the importance of discharge planning upon entering the medical profession, namely, by integrating this topic into the medical school education curriculum in Taiwan [16].

A European study including five countries reported that hospital physicians think that discharge is often delegated to nurses or junior physicians [17]. Lack of a standard discharge consultation was also found. In Taiwan, most senior physicians have not studied the discharge planning curriculum. They learn it by practice and there is no standard curriculum. In our study, we found senior attending physicians do not put effort into discharge planning and consider it as the nurses’ duty. Such thinking lets the junior physicians not focus on discharge planning. This may also alter the physicians’ role in discharge planning [18].

The PGY’s primary supervisor is the attending physician, who is also the primary decision maker for any disposition or treatment for a patient. Thus, the attitude of the attending physician has a major impact on PGY participants. Conflicts may also arise, but PGY participants respect their teachers and keep these doubts and conflicts to themselves. These problems have gradually emerged during the implementation of discharge planning. Since it is a national and hospital policy, physicians tend to implement it and communicate with each other. Now, as Taiwan enters an aging society, the beliefs should change.

### Subspecialty training system

In the hospital, healthcare providers do not sufficiently prioritize discharge consultations with patients and family members due to time restraints and competing care obligations [17]. In Taiwan, due to the priority of daily distribution of work, physicians are unable to focus on stable patients to be discharged. As junior physicians in the hospitals, PGY physicians worry about lack of experience and sometimes feel overburdened. The subspecialty training system makes them think residents are only responsible for hospitalization. Residents’ end-of-rotation transitions cause patients’ longer length of hospitalized stay [19] and higher mortality risk in internal medicine inpatient care [20]. The structure of hospital physicians’ and nurses’ work shifts restrict the care of discharge planning.

### Daily work overload

In the policy integrated system, PGY physicians should also utilize telephone follow up (TFU) after the patient discharged. Some physicians felt work overload. However, TFU can reduce hospital readmission within 30 days for patients with chronic disease [21]. We asked PGY physicians perform the TFU when the patient needs it, and the PGY physician can communicate with the patient when they return home. In the original system, they do not contact the patient after discharge.

It is challenging for a hospital physician with a heavy patient load to prepare discharge planning prior to the hospital discharge [22]. Coit also found that decreasing resident workload can improve discharge summary quality [23]. PGY physicians in Taiwan have the upper limit of patient load. Therefore, in our study, residents learn better when under less pressure during PGY training.

In addition to the above findings, our article also pointed out two advantages: the first is the participants’ improvement, and the second is the policy integrated discharge planning system.

### Participants’ improvement

In this study, we choose PGY physicians to teach discharge planning programs. At first, PGY physicians alerted us that they lack experience. They learned the identification of discharge planning needs, self-identification within the team, the improvement relationship between team members and the physician–patient relationship through practice.

Through the study, PGY physicians came to know more about other roles, although passively; this improved the division of labor and the relationship between team members. In a previous study, unclear role classification altered the motivation of health care workers about discharge planning [4]. In our study, the situation became better because it did increase PGY physicians’ identification of discharge planning needs, self-identification within the team, and the relationship between team members.

Through discharge planning situated learning, the trainer can learn interprofessional activity positively toward teamwork [24]. Frequent communication on role identities could prevent the misunderstanding of job tasks. Discharge planning is important due to its impact on patient outcomes, and can demonstrate the critical role teams play in interprofessional teamwork [25]. Our study, which emphasized learning by doing, could provide such opportunities for PGY physicians to study interprofessional teamwork.

### Policy integrated discharge planning system

For the final objective, our evaluation revealed that government pay-for-performance programs policies can enhance the clinical learning environment. In 2013, Wolk et al. found that pay-for-performance programs for time-to-discharge summary dictation could be an effective strategy for improving the behavior of medicine residents [26]. In our study, the policy integrated system included pay-for-performance programs for discharge planning, and also supported the hospital and physicians to promote discharge planning curriculum. Without government financial support and policies being enforced, the physicians’ attitude is passive.

Hospitals are emphasizing the implementation of discharge planning, making it imperative for physicians to understand and implement this program. These policies can help hospital decision-makers and physicians recognize the importance of discharge planning and the physician's role in it.

One of the challenges for medical educators is the changing nature of their research object—how to educate a continuous learning and responsive physicians to the changing healthcare system—and this study contributes to this challenge based on a case study on discharge planning with the CHAT framework. The solution lies in a dual effort: medical educators should not only decrease the inconsistencies identified in the activity, but also utilized the advantages found in the activity. Although we are not trying to generalize the discussion based on discharge planning training to cover other healthcare training issues, medical educators might take a page out of our book for this challenge in medical education.

Besides, the CHAT framework and Change Laboratory approach contribute to our study in two different ways. One is analytical, the other is contractive. The findings in Result section, which includes six elements, four contradictions, and four improvements, are belonged to the analytical contributions from the theoretical perspective, while the arguments in the most part of Discuss section, which includes three inconsistencies and two advantages, are belonged to the contractive contributions. These two distinct implications respectively help our study. Analytical contributions help us review six elements to find out the problems (contradictions) and potential (improvements) at the same time, while contractive contributions elevate our study based on the discussions on inconsistencies and advantages to a wider dialogue with the challenges in medical education.

Lastly, we should address some limitations of our research. One is the follow-up situations of PGY’s improvement. There is a 3–4 year Resident training next to the 2- year PGY, which imply a risk of losing holistic view during specialty training. Even our study has confirmed the discharge planning for GPY training is desirable and feasible in medical education, the follow-up situations from R to attending physician are needed to be taken into consideration. The other is our findings’ generalizability to other healthcare system. Taiwan is a high population density country, and our study also based on one medical center of Northern Taiwan. Therefore, it would limit us to generalize into the healthcare systems located in areas or countries with different level of population density.

## Conclusions

Discharge planning allows physicians who have completed PGY training to understand the big picture of care. This study aims to help PGY participants understand the importance of continuity of care and teamwork through discharge planning training, increase their self-identification as physicians, and educate the next generation of physicians about discharge planning.

With the full implementation of the PGY system in Taiwan, it is time for PGY participants to learn the importance of discharge planning. This study used the teaching and implementation of the discharge planning program as a core theme of the Change Laboratory and identified four benefits of using CHAT: participants learn through practice to improve (1) their identification of discharge planning needs, (2) their team self-identification, (3) the relationship between team members, and (4) the physician–patient relationship.

This has implications for patients’ medical habits and for the direction of medical practice and medical education. With time, long-term care has become more important, and physicians’ thinking and training must be updated. Physicians need to be able to identify the needs of their patients after discharge and make connections to help them and their families. This is what this study and curriculum will bring to physicians.

The CHAT and the Change Laboratory are designed to examine actual practices of PGY participants in discharge planning and to identify contexts, difficulties, and opportunities. It is also expected that additional studies will be conducted to improve the teaching of discharge planning among physicians. For example, how do medical educators implement the teaching of discharge planning in resident training stage as a common competency is a potential area for future research. Another important and related research issue is to develop core competency, milestone, and entrustable professional activity (EPA) about discharge planning, whose formulate can be integrated into national policies in medical practice.

In the context of discharge planning, our research based on Taiwan experience might be related to global trends in two specific ways. One is the common challenges brought by so-called ‘aged or super aged society’ trends. Discharge planning’s importance will raise with the aging degree of population, and Taiwan would not stay out of it. The other one is specific challenge: the family-prioritized care culture of East Asian countries. The role of caregiver is often assigned to family members, especially younger, female, those without paid work. Discharge planning in Taiwan always need to face the problems, such as resist to day care centers, skilled nursing facilities, or hospice agencies. Taking this kind of care culture into consideration is a necessary cultural factor in East Asian area.

## Data Availability

The datasets and/or analyzed during the current study available from the corresponding author on reasonable request due to privacy concerns.
